# Severe ulcerative colitis induced by COVID-19 vaccination

**DOI:** 10.1007/s12328-024-01926-x

**Published:** 2024-02-13

**Authors:** Takashi Taida, Jun Kato, Kentaro Ishikawa, Naoki Akizue, Yuki Ohta, Kenichiro Okimoto, Keiko Saito, Keisuke Matsusaka, Tomoaki Matsumura, Naoya Kato

**Affiliations:** 1 Department of Gastroenterology, Graduate School of Medicine, Chiba University, Chiba, Japan; 2 Endoscopic Center, Chiba University Hospital, Chiba, Japan; 3 Department of Gastroenterology, Chiba Rosai Hospital, Chiba, Japan; 4 Department of Pathology, Chiba University Hospital, Chiba, Japan

**Keywords:** COVID-19, Vaccine, Inflammatory bowel disease

## Abstract

A 37-year-old woman developed severe colitis with diffuse mucosal erythema and ulcerations throughout the entire colon after the 3rd vaccination of COVID-19. Stool culture was negative, and the pathological findings showed increased lymphoplasmacytic and neutrophilic infiltration in the colonic lamina propria, which were consistent with ulcerative colitis. After the treatment with anti-tumor necrosis factor-α agent, the ulceration markedly improved with development of severe colonic stenosis, which was successfully dilated with endoscopic balloon dilation. In case of COVID-19 vaccination, it should be noted that vaccination could be a trigger for the onset of UC.

## Introduction

COVID-19 is an infectious disease caused by the SARS-CoV-2 virus. It is highly contagious and can cause mild to severe respiratory illness. The vaccination against the virus can cause adverse events, although the benefits of vaccination are considered to outweigh the potential risks. Here, we report a patient who developed severe ulcerative colitis (UC) just after the vaccination for COVID-19. The present case seems rare but represents an important adverse event caused by COVID-19 vaccine.

## Case report

**Fig. 1:**
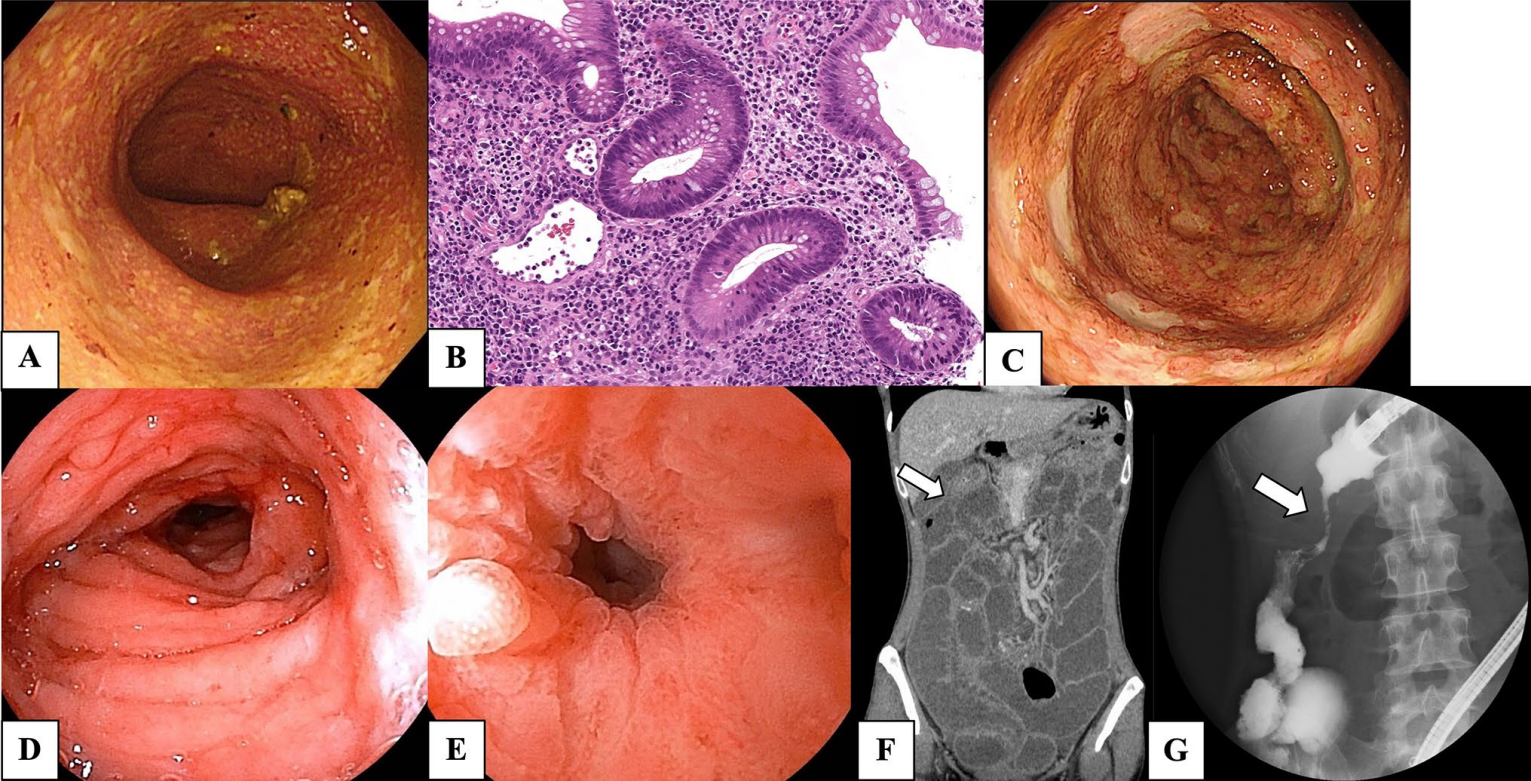
Endoscopic, histological, and radiologic images of the case. Rectum on the ninth day after COVID-19 vaccination (**A**). Histology of the biopsy specimens from the rectum (**B**). Sigmoid colon on the 23th day after vaccination (**C**). Sigmoid colon (**D**) showed marked improvement of inflammation but stenosis was observed at the transverse colon (**E**), after the administration of infliximab. Abdominal CT scan (**F**) and radiologic image at colonoscopy (**G**) showed severe stenosis at the hepatic flexure (arrow)

**Fig. 2:**
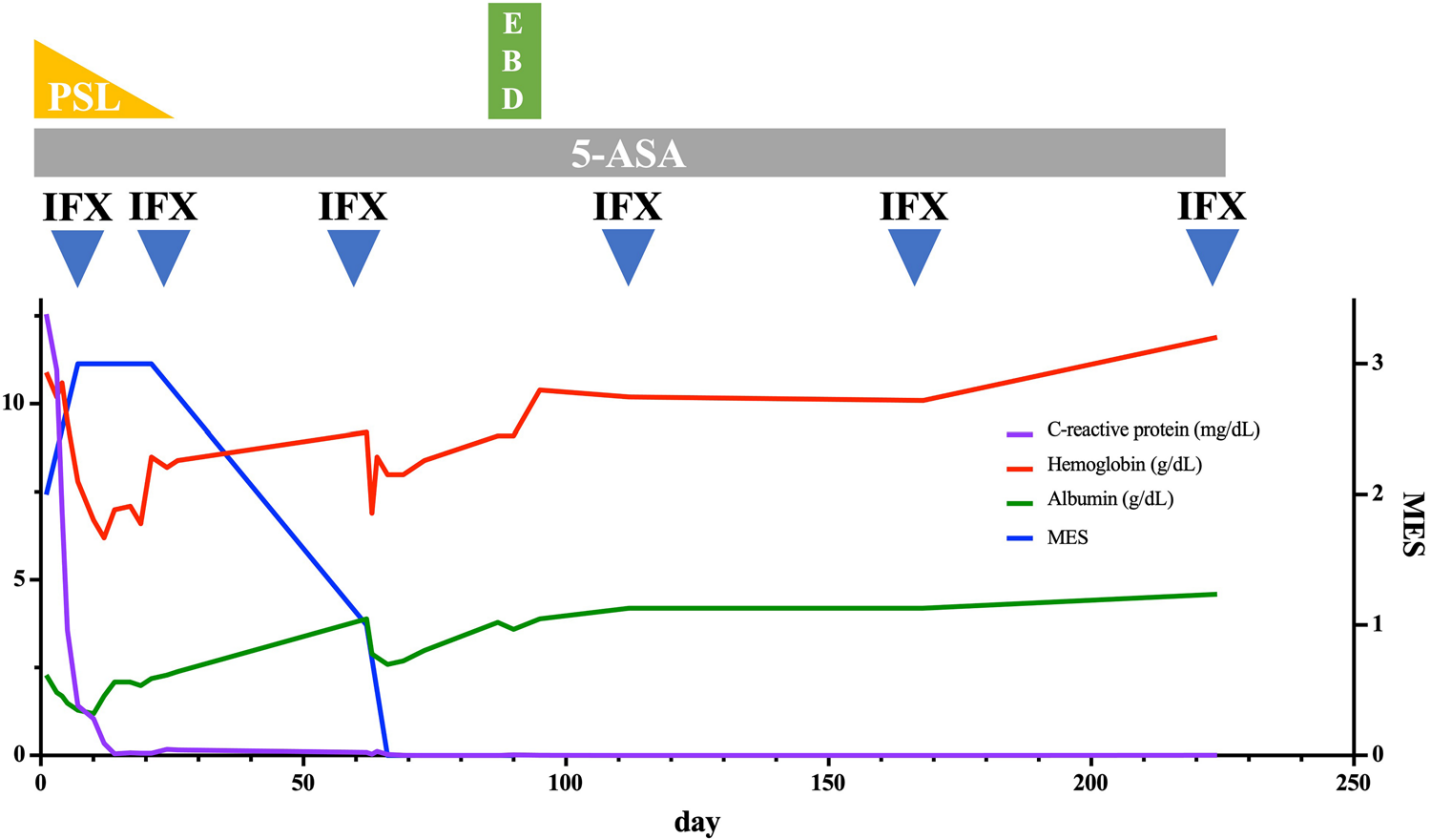
Clinical course of the case. 5-*ASA* 5-aminosalicylic acid, *EBD* endoscopic balloon dilatation, *MES* Mayo endoscopic subscore, *IFX* infliximab, *PSL* prednisolone

A 37-year-old woman developed nausea, abdominal pain, and severe diarrhea approximately 12 h after the administration of the third dose of COVID-19 vaccine (Pfizer-BioNTech-Comirnaty). She visited our hospital nine days after the onset of these symptoms. A laboratory test showed a highly elevated C-reactive protein (CRP) of 12.56 mg/dL. In addition, serum albumin was decreased to 2.3 g/dL (Table 1). The stool culture was negative. The CMV antigen was negative. Sigmoidoscopy revealed edematous mucosa with extensive erythema and ulceration throughout the observation range (Fig. 1A). Histology of the biopsy specimens from the rectum showed crypt distortion, glandular atrophy, loss of goblet cells, and lymphoplasmacytic inflammation in lamina propria with plasma cells in base of mucosa (basal plasmacytosis), indicating chronic and ongoing mucosal injury (Fig. 1B). Based on these findings, the patient was diagnosed as UC. Although 5-aminosalycylate and subsequent corticosteroids were administered, the patient was resistant to these treatments (Fig. 1C). After the induction with infliximab, an anti-tumor necrosis factor (TNF)-α antibody, the patient achieved clinical and mucosal remission (Fig. 1D). Two months after the initiation of infliximab, the patient developed a bowel obstruction. Abdominal CT scan and colonoscopy showed severe stenosis at the hepatic flexure (Fig. 1E-G). Biopsy specimens obtained around the stenosis showed negative findings of malignancy or granuloma formation, and endoscopic balloon dilation for the stenosis was performed with successful scope passage. After endoscopic dilatation of the colon, no evidence of lesions in the small intestine was confirmed with enteroscopy and small bowel follow-through. The patient has maintained remission with infliximab and had no recurrent bowel obstruction (Fig. 2).

**Table 1:** Results of laboratory investigations on the first visit

Complete blood count		
WBC	12,600	/μL
RBC	383	×10^4^/μL
Hb	10	g/dL
Hct	33.2	%
MCV	86.7	fL
MCH	28.5	pg
MCHC	32.8	%
Plt	43.9	×10^3^/μL
MPV	9.4	fL
Blood smear		
Stab	33.5	%
Segmented	53.5	%
Lymphocyte	6.5	%
Monocyte	4	%
Eosinophil	0	%
Basophil	0	%
Coagulation		
PT(s)	19.4	s
PT(%)	51	%
APTT(s)	43	s
D-dimer	8.4	μg/mL
Biochemistry		
TP	5.7	g/dL
Alb	2.3	g/dL
AST	16	IU/L
ALT	14	IU/L
LDH	146	IU/L
ALP	54	IU/L
γGTP	11	IU/L
BUN	4.5	mg/dL
Cre	0.5	mg/dL
Na	135	mmol/L
K	2.7	mmol/L
Cl	96	mmol/L
T-Bill	0.6	mg/dL
CRP	12.56	mg/dL
FER	115	ng/dL
Fe	8	μg/dL
Immunology		
CMV pp65 antigen	(-)	

*Alb* albumin, *ALP* alkaline phosphatase, *ALT* alanine aminotransferase, *APTT* activated partial thromboplastin time, *AST* aspartate aminotransferase, *BUN* blood urea nitrogen, *CMV* cytomegalovirus, *Cre* creatinine, *CRP* C-reactive protein, *FER* feritin, *γGTP* gamma-glutamyl transferase, *Hb* hemoglobin, *Hct* hematocrit, *LDH* lactate dehydrogenase, *MCH* mean corpuscular hemoglobin, *MCHC* mean cell hemoglobin concentration, *MCV* mean corpuscular volume, *Plt* platelets, *PT* prothrombin time, *RBC* red blood cells, *T-Bil* total bilirubin, *TP* total protein, *WBC* white blood cells

## Discussion

According to the World Health Organization (WHO) Coronavirus disease situation dashboard, as of May 9, 2023, there have been 765,903,278 confirmed cases of COVID-19 and 6,927,378 deaths reported worldwide. Vaccines are available to prevent COVID-19 and have been shown to be safe and effective. According to a COVID-19 Results Briefing by the Institute for Health Metrics and Evaluation, 82% of people in Japan have received at least one vaccine dose, and 78% have received two or more vaccine doses.

The vaccine provides an essential benefit in preventing the fatal course caused by COVID-19, whereas the vaccination can cause adverse events. Injection site events (e.g., pain, redness, swelling) and systemic effects (e.g., fatigue, headache, muscle or joint pain) were reported in randomized clinical trials of COVID-19 vaccines, with rare serious adverse events [1]. Serious adverse events of special interest were observed in mRNA COVID-19 vaccine trials, with an excess risk of 12.5 per 10,000 vaccinated [2]. In that report, the incidence of colitis was 0.5 per 10,000 patients in both the vaccine and placebo groups, with no difference between the two groups.

Nonetheless, the present case developed UC immediately after the COVID-19 vaccination. Although it is difficult to prove a causal relationship with the vaccine, the patient had no symptoms of colitis before vaccination, with no evidence of the other gastrointestinal diseases, and therefore, the influence of vaccination on the development of UC cannot be dismissed.

The emerging evidence has indicated that COVID-19 vaccination is associated with new onset of autoimmune manifestations including thrombotic thrombocytopenia, autoimmune liver disease, Guillain–Barre syndrome and IgA nephropathy [3]. As a mechanism by which vaccination was involved in autoimmune response, it is assumed that the cross-reactivity of proteins present in SARS-CoV-2 with various tissue antigens of the host induces autoimmunity against connective tissue, cardiovascular, gastrointestinal, and nervous systems.

Regarding gastrointestinal disease, there have been a few reports showing a case with the development of UC after COVID-19 vaccination. One report described the development of UC after a second vaccination with MVC-COVI1901, the protein subunit vaccine [4]. The MVC-COVI1901 vaccine targets S-2P, as well as the Modela COVID-19 vaccine (Spikevax), and the vaccine-induced expression of S-2P activates Toll-like receptor (TLR) 4. In addition, the adjuvant contained in the vaccine also activates TLR9 and the expression of interleukin-13. The combination of these two factors could contribute to the development of UC. Although Comirnaty administered in the present case was designed to target a different viral spike protein from S-2P, and it was reported that induction of Th17 cells with Comirnaty was slight[5], the viral spike protein induced by this vaccine can also function similarly to the MVC-COVI1901 vaccine. Therefore, this vaccine can induce immunostimulation on the host intestinal immune system through activation of TLRs, and trigger the development of UC. In this context, it has been reported that COVID-19 vaccination for patients with inflammatory bowel disease (IBD) can induce flare of the disease [6], although a recent report did not show the significant increase in flare due to vaccination [7]. In a multicenter observational controlled study conducted in Japan, 5.2% of patients with IBD had flares at 4 weeks after vaccination, and some healthy controls also developed diarrhea after vaccination [8].

Although there was a report about the onset of UC after the first vaccination with Comirnaty, the reported patient improved with the administration of corticosteroids, followed by maintenance therapy with 5-ASA [9]. In contrast, the present case had a steroid-refractory course, which required induction with an anti-TNF-α agent. Vedolizumab was used for both remission induction and maintenance in the case of UC caused by another COVID vaccine who was resistant to corticosteroids [4]. Appropriate remission induction and maintenance therapies are required in vaccine-induced UC cases as in conventional UC.

Serious colitis requiring biologics and inducing stenosis may be rarely caused by COVID-19 vaccination. However, evidence that the vaccination could trigger the onset of IBD is still scarce. Further research is needed to better understand the potential risks for the onset of IBD and benefits of COVID-19 vaccination.
